# Associations between school enjoyment at age 6 and later educational achievement: evidence from a UK cohort study

**DOI:** 10.1038/s41539-021-00092-w

**Published:** 2021-06-15

**Authors:** Tim T. Morris, Danny Dorling, Neil M. Davies, George Davey Smith

**Affiliations:** 1 MRC Integrative Epidemiology Unit at the University of Bristol, Bristol, UK; 2 Population Health Sciences, Bristol Medical School, University of Bristol, Bristol, UK; 3 School of Geography and the Environment, University of Oxford, Oxford, UK; 4 K.G. Jebsen Center for Genetic Epidemiology, Department of Public Health and Nursing, NTNU, Norwegian University of Science and Technology, Trondheim, Norway

**Keywords:** Education, Sociology, Human behaviour

## Abstract

Education is influenced by a broad range of factors but there has been limited research into the role that early school enjoyment plays in pupil’s educational achievement. Here we used data from a UK cohort to answer three research questions. What is the association between early school enjoyment and later academic achievement? To what extent do family background factors underlie this association? Do sex differences in school enjoyment underlie sex differences in achievement? School enjoyment was self-reported in two questionnaires completed at age 6. We used multiple imputation to account for missing covariates in this study, giving an imputed sample size of 12,135. Children’s school enjoyment at age 6 associated with sex and cognitive ability but not family socioeconomic background. For example, girls were twice as likely to report enjoying school than boys (OR: 1.97; 95% CI: 1.56, 2.48). School enjoyment strongly associated with later achievement in age 16 compulsory GCSE exams even after adjustment for socioeconomic background and cognitive ability; pupils who reported enjoying school scored on average 14.4 (95% CI: 6.9, 21.9) more points (equivalent to almost a 3-grade increase across all subjects) and were 29% more likely to obtain 5 + A*-C GCSE’s including Maths and English (OR: 1.29; 95% CI: 0.99, 1.7) than those who did not enjoy school. These results highlight the importance of school enjoyment for educational achievement. As a potentially more modifiable factor than socioeconomic background, cognitive ability or sex, school enjoyment may represent a promising intervention target for improving educational outcomes.

## Introduction

Educational achievement is influenced by a vast array of factors including cognitive ability^1^; teacher ability^2,3^; school quality^4,5^; parental academic support^6^; family socioeconomic background^7,8^; and peers^9^. One area where there is relatively little evidence is school enjoyment^10^, despite its ‘common sense’ link to achievement^11^. It has been acknowledged that enjoyment of learning is a key aim for educators and policymakers to improve pupils’ educational experience and outcomes^12^, and school enjoyment is, at least intuitively, far more easily modifiable than other factors such as family socioeconomic position^11^. School enjoyment may therefore offer a promising target for interventions to increase educational achievement.

Prior research has demonstrated that children who enjoy school are much more likely to outperform those who dislike school^13–15^. In a study of 90 Chicago high school students’ performance in a research project, enjoyment of the project was the second strongest predictor of final grade (behind high school grade point average) and accounted for almost half a grade difference^13^. In a study of 388 psychology university students in Austria, exam enjoyment was associated with a one-sixth increase in final grade where grades were scaled from 1 to 5^15^. A meta-analysis of academic boredom and academic outcomes (motivation, learning strategies and achievement) revealed an overall correlation of −0.24^14^. Studies have also observed an association between enjoyment of specific school subjects such as Maths and reading and higher subject performance^16–18^. In an analysis of the Longitudinal Survey of Young People in England (LSYPE), Vignoles and Meschi found that amongst a sample of ~11,000 children, school enjoyment at age 14 associates strongly with achievement at age 16^19^. Sylva et al.^20^ observed positive associations between school enjoyment at age 11 and achievement at age 14 that are stronger for Mathematics than English. The inconsistent measurement of enjoyment hinders the comparability of these results, but the overall trend of findings suggests that enjoyment of the learning environment is strongly and positively associated with achievement. However, there is some inconsistency in study findings. Two studies investigating school enjoyment during primary school years found no strong evidence that school enjoyment associated with contemporaneous achievement^20,21^, but associations with later achievement were not analysed.

School enjoyment has been hypothesised to result in higher academic inspiration and motivation to learn^11^, which in turn leads to higher educational achievement^22^. Enjoyment is a positive emotion beneficial to wellbeing that can promote learning^23^. School-based interventions can promote positive emotions such as enjoyment^24,25^ which could in turn lead to higher levels of academic achievement and wellbeing^10,26^. However, much of the literature focuses on school enjoyment in mid and late childhood. Children who perceive themselves as having low ability are less likely to enjoy school or education activities^27,28^ and school enjoyment at later ages may therefore in part reflect earlier achievement^19^. Using measures of school enjoyment assessed at ages 7 and 10, Gutman and Vorhaus demonstrated strong associations between school enjoyment and engagement, which they argue as “indicating that children who enjoy school are more likely to be motivated and engaged in their school work at a later point in time”^29^. School enjoyment later in childhood could therefore reflect feedback effects of reward systems from achievement earlier in childhood and arise from a reaction to external reward factors that high ability pupils experience rather than reflecting intrinsic motivations^30^. From the receiving of awards, through to being allocated to high ability sets, there are numerous ways in which the behaviour of children who act in certain ways is rewarded at schools in a manner that might lead some to feel greater satisfaction. Conversely, the imposition of punishments such as detention for some behaviours through to subtle but public chastisement of a child by a teacher in a classroom are examples of how children who do worse at school can be made to feel worse too. Earlier measures of school enjoyment that are more robust to such feedback effects may provide stronger evidence about the effects of school enjoyment on later achievement and better indication of the potential for early interventions. Furthermore, earlier measures of school enjoyment are also appealing as they offer the longest period of potential intervention before later education and testing outcomes and could therefore offer the most promising point for interventions.

Gutman and Vorhaus provided an overview of the ways in which dimensions of childrens, wellbeing (emotional, behavioural, social and academic) associate with their educational achievement^29^. They highlight that many previous studies have combined school enjoyment with other measures of school wellbeing or as an input to constructs of positive emotions (c.f.^15,28^) that prohibits disentangling the unique effects of children’s school enjoyment on their outcomes. Previous research has found that school support is positively associated with school enjoyment while mental health-related needs are negatively associated^11^. Furthermore, few studies have appropriately controlled for parental or family factors as potential confounders. This reflects a general lack of understanding of the family level drivers of school enjoyment. It has been highlighted how many previous studies have failed to appropriately account for background family factors due to limitations of administrative data^19^, so the extent to which a pupils, school enjoyment reflects their family social background is unknown. Relationships with teachers and peers, pupil temperament and attitude to learning, pupil confidence in ability and perceived quality of work, classroom activities, teachers, attitude and school facilities are also important determinants of school enjoyment^12,31–33^. Each of these are linked in complex ways to family background characteristics through the social structures surrounding UK education. It has been observed that a pupils, school enjoyment is unrelated to the school that they attend^19,34^. School enjoyment therefore may be considered to reflect something inherent to a child or their upbringing rather than due to extraneous school contextual factors.

Girls have been estimated to have higher levels of school enjoyment than boys^35^ and there is evidence that school enjoyment declines more rapidly amongst girls from primary to secondary school than amongst boys^36,37^. However, this may not hold true across all aspects of schooling and there will be considerable variability between individual children. For example, one study found evidence that boys were more likely to enjoy maths lessons than girls^16^. There is also some evidence that school enjoyment may mediate sex differences in achievement^17^, raising the possibility that enjoyment plays a role in the formation of sex inequalities in education. As with other drivers of educational achievement, difficulties arise in isolating the contribution that school enjoyment makes independent of other factors. The substantial socioeconomic inequalities that pervade education in the UK could confound associations^7,38–40^.

Whether the association of school enjoyment and achievement is confounded by factors such as family socioeconomic position has important implications for teachers and policy makers. For example, a child’s enjoyment of school may partly reflect parental involvement or interest in their education, which is itself highly socially patterned^41^. Previous studies have focussed on enjoyment at the time of examination^13–15^, but enjoyment may vary considerably throughout a child’s schooling, from initial enrolment through to final examinations. Strong longitudinal associations between school enjoyment in early childhood and later achievement may offer promise for early intervention possibilities before educational inequalities can widen with age^7,38,40^. Thus, there is limited longitudinal evidence about the relationship between school enjoyment and academic achievement.

In this study we used data from a UK cohort study, the Avon Longitudinal Study of Parents and Children (ALSPAC), to answer the following research questions. (1) What is the association between early school enjoyment and later academic achievement? (2) To what extent do family background factors underlie this association? (3) Do sex differences in school enjoyment underlie sex differences in achievement? We first assessed the socioeconomic patterning of self-reported school enjoyment in early childhood to determine if this could confound enjoyment-achievement associations. We then test the association between early school enjoyment and educational achievement at age 16 controlling for a range of potential confounding factors including parental education, socioeconomic position and cognitive ability to investigate if these explain associations between school enjoyment and achievement. Finally, we test whether sex differences in enjoyment help to explain the sex gap in educational achievement.

## Results

### Descriptive statistics

Of the 12,135 ALSPAC participants who were alive at 1 year of age and had data on biological sex at birth, month of birth and ethnicity, 9591 participants had at least one data value missing on school enjoyment, academic achievement and all covariates, giving a complete case sample of 2544. Item missingness was generally low; half of all participants had fewer than 3 missing values of the 14 variables of interest (Fig. S2). To overcome item-specific missingness and reduce potential selection bias in the complete case sample^42^, we performed multiple imputation (see Methods section). The descriptive statistics of the imputed sample (*n* = 12,135) were highly similar to that of the complete case sample (Table 1). School enjoyment was measured using two questionnaires completed by ALSPAC participants when they were aged six (see Methods section). In all, 78% of the imputed sample reported enjoying school at both ages, 16% enjoyed school at 1 age, and 6.3% did not enjoy school at either age. Compared to the multiple imputed dataset, the complete case dataset resulted in underrepresentation of non-white participants, families with parents in more routine and manual occupations, families with less educated mothers and children who scored lower on cognitive ability tests from the wider ALSPAC cohort.

**Table 1:** Descriptive statistics from complete case and multiple imputation samples.

	Pre-imputation (*n* = 2544)		Missingness	Post-imputation (*n* = 12,135)	
	*N* (mean)	% (SD)	*N* (%)	*N* (mean)	% (SD)
GCSE points	(358.51)	(70.17)	2126 (18)	(326.22)	(89.47)
Enjoys school			6816 (56)		
No enjoyment	142	5.58	–	762	6.28
Mixed enjoyment	363	14.27	–	1940	16.0
Enjoyed school	2039	80.15	–	9433	77.7
Sex			0 (0)		
Male	1160	45.6	–	6254	51.5
Female	1384	54.4	–	5881	48.5
Month of birth			0 (0)		
Sep	242	9.51	–	1191	9.81
Oct	266	10.46	–	1173	9.67
Nov	227	8.92	–	1114	9.18
Dec	218	8.57	–	993	8.18
Jan	153	6.01	–	681	5.61
Feb	106	4.17	–	568	4.68
Mar	138	5.42	–	709	5.84
Apr	213	8.37	–	962	7.93
May	256	10.06	–	1099	9.06
Jun	241	9.47	–	1165	9.60
Jul	246	9.67	–	1288	10.61
Aug	238	9.36	–	1192	9.82
Ethnicity			0 (0)		
White	2460	96.7	–	11,524	94.97
Non-white	84	3.3	–	611	5.04
Social class			815 (7)		
IV & V	420	16.51	–	2485	20.47
III Manual	624	24.53	–	3573	29.44
III Non-manual	706	27.75	–	3001	24.73
I & II	794	31.21	–	3076	25.35
Maternal education			67 (1)		
CSE/vocational	454	17.85	–	3525	29.05
O-level	912	35.85	–	4245	34.98
A-level	740	29.09	–	2775	22.87
Degree	438	17.22	–	1590	13.10
IQ	(0.17)	(0.95)	5453 (45)	(−0.12)	(1.02)
School year			1621 (13)		
2006/2007	535	21.03	–	2562	21.11
2007/2008	1567	61.6	–	7413	61.09
2008/2009	442	17.37	–	2160	17.80
Likes teacher			4108 (34)		
Not at all	12	0.47	–	109	0.90
Sometimes	101	3.97	–	538	4.44
Usually	698	27.44	–	3566	29.39
Always	1733	68.12	–	7920	65.27
Home learning environment	(7.05)	(1.05)	2648 (22)	(6.88)	(1.22)
Temperament	(17.84)	(1.91)	6196 (51)	(17.84)	(1.88)
Work confidence	(11.83)	(3.56)	5082 (42)	(12.65)	(1.78)
Intelligence confidence	(8.08)	(2.48)	5084 (42)	(7.63)	(1.71)
Friends score	(3.38)	(2.3)	5779 (48)	(3.44)	(2.42)

Percentages may not sum to 100 due to rounding.

*GCSE* General Certificate of Secondary Education, *CSE*Certificate of Secondary Education.

### Is school enjoyment socioeconomically or demographically patterned?

There was no clear social patterning of school enjoyment by parental socioeconomic position based upon occupation class or mothers, highest level of education (Table 2). Children with parents in more skilled occupations were as likely to report enjoying school as those with parents in less skilled and routine occupations. Children with more highly educated mothers were generally as likely to report enjoying school as those with less educated mothers, though children with degree-educated mothers were more likely to report enjoying school at both occasions (Odds ratio: 1.36; 95% CI: 0.99, 1.87). Children with a one standard deviation higher measured cognitive ability as measured at age 8 (see Methods section) were also more likely to say they enjoyed school than did not enjoy school (OR: 1.24; 95% CI: 1.11, 1.38). After adjustment for all covariates, there remained strong evidence of an association between cognitive ability and school enjoyment (OR: 1.16; 95% CI: 1.02, 1.32). There was little evidence of two-way interactions between each of social class, maternal education and cognitive ability. Girls were twice as likely to say they enjoyed school than boys after adjustment for all covariates (OR: 1.97; 95% CI: 1.56, 2.48) (Supplementary Table 2). Non-white children were almost twice as likely to report enjoying school than white children (OR: 1.87; 95% CI: 0.99, 3.55). There was strong evidence that children born later in the school year were less likely to enjoy school than those born earlier in the school year (OR: 0.95; 95% CI: 0.92, 0.99), though this association attenuated after adjustment for all covariates (OR: 0.98; 95% CI: 0.94, 1.01). There was strong evidence for large associations between a child’s opinion of their teacher and their school enjoyment; children who reported the strongest relationships with their teachers were over nine times as likely to report enjoying school than those who did not like their teacher (OR: 9.44; 95% CI: 4.45, 20.02). Children who self-reported more positive and friendly temperaments were also more likely to report enjoying school than children who reported more negative or angry temperament (OR: 1.19; 95% CI: 1.12, 1.26), and children who reported higher confidence in the quality of their work were more likely to report enjoying school (OR: 1.36; 95% CI: 1.27, 1.45). Associations were consistent across the imputed and complete case datasets, though there was far less precision in the complete case analyses (Supplementary Tables 2 and 3).

**Table 2:** Multinomial regression of school enjoyment on key parameters in multiple imputation sample. Full parameter estimates provided in Supplementary Table 2.

	Independent associations			Fully adjusted associations		
	OR	95% CI	*p* value	OR	95% CI	*p* value
No enjoyment	Ref			Ref		
Mixed enjoyment						
Social class						
IV & V	Ref	–	–	Ref	–	–
III Manual	0.93	0.69, 1.26	0.633	0.89	0.65, 1.21	0.450
III Non-manual	1.01	0.72, 1.41	0.954	0.93	0.66, 1.32	0.692
I & II	0.98	0.72, 1.35	0.919	0.87	0.61, 1.25	0.463
Maternal education						
CSE/vocational	Ref	–	–	Ref	–	–
O-level	1.12	0.86, 1.47	0.397	1.12	0.86, 1.47	0.397
A-level	0.98	0.73, 1.31	0.877	0.98	0.73, 1.31	0.877
Degree	1.30	0.91, 1.85	0.145	1.30	0.91, 1.85	0.145
Cognitive ability	1.08	0.97, 1.20	0.141	1.03	0.91, 1.17	0.604
Enjoyed school						
Social class						
IV & V	Ref	–	–	Ref	–	–
III Manual	0.99	0.76, 1.29	0.941	0.90	0.67, 1.19	0.450
III Non-manual	1.16	0.87, 1.55	0.305	0.98	0.71, 1.34	0.886
I & II	1.09	0.82, 1.47	0.55	0.87	0.61, 1.24	0.443
Maternal education						
CSE/vocational	Ref	–	–	Ref	–	–
O-level	1.15	0.91, 1.46	0.251	1.09	0.84, 1.41	0.526
A-level	1.12	0.86, 1.45	0.411	1.04	0.77, 1.41	0.778
Degree	1.36	0.99, 1.87	0.060	1.33	0.90, 1.96	0.156
Cognitive ability	1.24	1.11, 1.38	<0.001	1.16	1.02, 1.32	0.025

*OR* odds ratio, *CI* confidence interval, *Ref* reference category, *CSE* Certificate of Secondary Education.

### School enjoyment and educational achievement

Table 3 displays the association of school enjoyment at age 6 and nationally standardised test results at age 16 (see Supplementary Table 4 for full parameter estimates). Achievement was measured as fine graded point scores from GCSE exams (see Methods section). Children who enjoyed school at age six scored on average 29.34 (95% CI: 19.4, 39.29) more points at GCSE and those had mixed enjoyment scored on average 12.53 (−2.42, 22.65) more points compared to children who did not enjoy school. Based on the calculation of 6 points per GCSE grade, these differences are equivalent to five and two grade differences in GCSE’s respectively. Controlling for family socioeconomic position and performance on cognitive ability tests attenuated these associations to 17.75 (10.45, 25.05) and 8.05 (−0.22, 15.89) respectively. Furthermore, controlling for all other covariates attenuated associations to 14.41 (6.9, 21.93) and 6.16 (−1.7, 14.02) point differences; broadly equivalent to differences of two and one grades. In the full model adjusting for all covariates, the association between enjoying school at age 6 and educational achievement at age 16 was almost as large as that for sex (*β*: 21.61; 95% CI: 18.73, 24.48) and the largest social class difference (*β*: 22.19; 95% CI: 17.49, 26.89). Maternal education had a much greater effect size on GCSE point scores than other variables, whereby children with degree educated mothers scored on average 43.84 (38.37, 49.31) points above children whose mothers had no qualifications, CSE or a vocational qualification. Measured cognitive ability was also strongly associated with GCSE score (*β*: 43.01; 95% CI: 41.25, 44.77), though care must be taken when interpreting this parameter as it was measured after school enjoyment. The results were generally consistent across the imputed and the complete case datasets (Supplementary Tables 4 and 5), though the complete case analyses overestimated associations between school enjoyment and achievement, and underestimated associations between both social background and cognitive ability with achievement relative to the estimates from the imputed analysis.

**Table 3:** Linear regression of age 16 GCSE achievement (capped points score) on key parameters in multiple imputation sample. Full parameter estimates provided in Supplementary Table 4.

	Model 1: unadjusted			Model 2: family socioeconomic position			Model 3: IQ adjusted			
	*β*	95% CI	*p* value	*β*	95% CI	*p* value	*β*		95% CI	*p* value
School enjoyment										
No enjoyment	Ref	–	–	Ref	–	–	Ref		–	–
Mixed enjoyment	12.53	2.42, 22.65	0.015	8.05	0.22, 15.89	0.044	6.16		−1.70, 14.02	0.125
Enjoyed school	29.34	19.4, 39.29	<0.001	17.75	10.45, 25.05	<0.001	14.41		6.90, 21.93	<0.001

Model 1 adjusts for sex, age in year, ethnicity, cohort year and age school enjoyment reported. Model 2 additionally adjusts for parental social class based on occupation and maternal education. Model 3 additionally adjusts for cognitive ability, home learning environment, opinion of teacher, temperament, the pupil’s confidence in their work, the pupil’s confidence in their intelligence and the perceived quality of their friendship group. Full parameter estimates presented in Supplementary Table 3.

*GCSE* General Certificate of Secondary Education, *CI* confidence interval, *Ref* reference category.

Results were similar in logistic regression analyses of obtaining 5 + A*-C GCSEs, including English and Maths; the required standard for many further educational and occupational opportunities (Table 4 and Supplementary Table 6 for full parameter estimates). Children who enjoyed school at age 6 were 64% more likely to obtain 5 + A*-Cs than children who did not enjoy school (OR: 1.64; 95% CI: 1.33, 2.03). Children with mixed enjoyment were also more likely to obtain 5 + A*-C GCSE’s than children who did not enjoy school, but the association was weaker (OR: 1.28; 95% CI: 1.02, 1.6). These associations were greater than sex differences in the odds of obtaining 5 + A*-C GCSE’s (OR for females compared to males: 1.50; 95% CI: 1.38, 1.62). The associations with levels of school enjoyment were attenuated after adjustment for socioeconomic position and cognitive ability (OR: 1.48; 95% CI: 1.16, 1.9 and OR: 1.27; 95% CI: 0.97, 1.66 respectively). The association of enjoyment and achievement was smaller than associations of occupational social class and maternal education with achievement. Controlling for all covariates further attenuated the association for children who enjoyed school at age 6 (OR: 1.29; 95% CI: 0.99, 1.67) and those who had mixed enjoyment (OR: 1.18; 95% CI: 0.9, 1.55). Performance in the cognitive ability test was the strongest predictor of achievement; a one standard deviation increase in cognitive ability was associated with over three times higher odds of obtaining 5 + A*-C GCSEs, (OR: 3.1; 95% CI: 2.88–3.34 s). The complete case analyses overestimated the association between school enjoyment and achievement but was otherwise consistent with the imputed dataset (Supplementary Tables 6 and 7).

**Table 4:** Logistic regression of age 16 GCSE achievement (achieving 5 + A*-C grades including English and Maths) on key parameters in multiple imputation sample. Full parameter estimates provided in Supplementary Table 6.

	Model 1: unadjusted			Model 2: family socioeconomic position			Model 3: IQ adjusted		
	OR	95% CI	*p* value	OR	95% CI	*p* value	OR	95% CI	*p* value
School enjoyment									
No enjoyment	Ref	–	–	Ref	–	–	Ref	–	–
Mixed enjoyment	1.28	1.02, 1.60	0.031	1.27	0.97, 1.66	0.081	1.18	0.90, 1.55	0.226
Enjoyed school	1.64	1.33, 2.03	<0.001	1.48	1.16, 1.90	0.002	1.29	0.99, 1.67	0.057

Model 1 adjusts for sex, age in year, ethnicity, cohort year and age school enjoyment reported. Model 2 additionally adjusts for parental social class based on occupation and maternal education. Model 3 additionally adjusts for cognitive ability, home learning environment, opinion of teacher, temperament, the pupils, confidence in their work, the pupils, confidence in their intelligence and the perceived quality of their friendship group. Full parameter estimates presented in Supplementary Table 4.

*OR* odds ratio, *GCSE* General Certificate of Secondary Education, *CI* confidence interval, *Ref* reference category.

Both linear and logistic models provided strong evidence for associations between the home learning environment at age 3 and achievement (*β*: 3.34; 95% CI: 2.06, 4.61; OR: 1.07; 95% CI: 1.03, 1.12), and between a child’s confidence in their intelligence and achievement at age 8 (*β*: 3.62; 95% CI: 2.65, 4.6; OR: 1.09; 95% CI: 1.05, 1.13). There was however no strong evidence for associations with achievement between childs, opinion of their teacher, temperament, confidence in work ability or the quality of their friendship group (all measured at age 6 or 8; see Methods section); these are either estimated to be small or with imprecision.

**Fig. 1:**
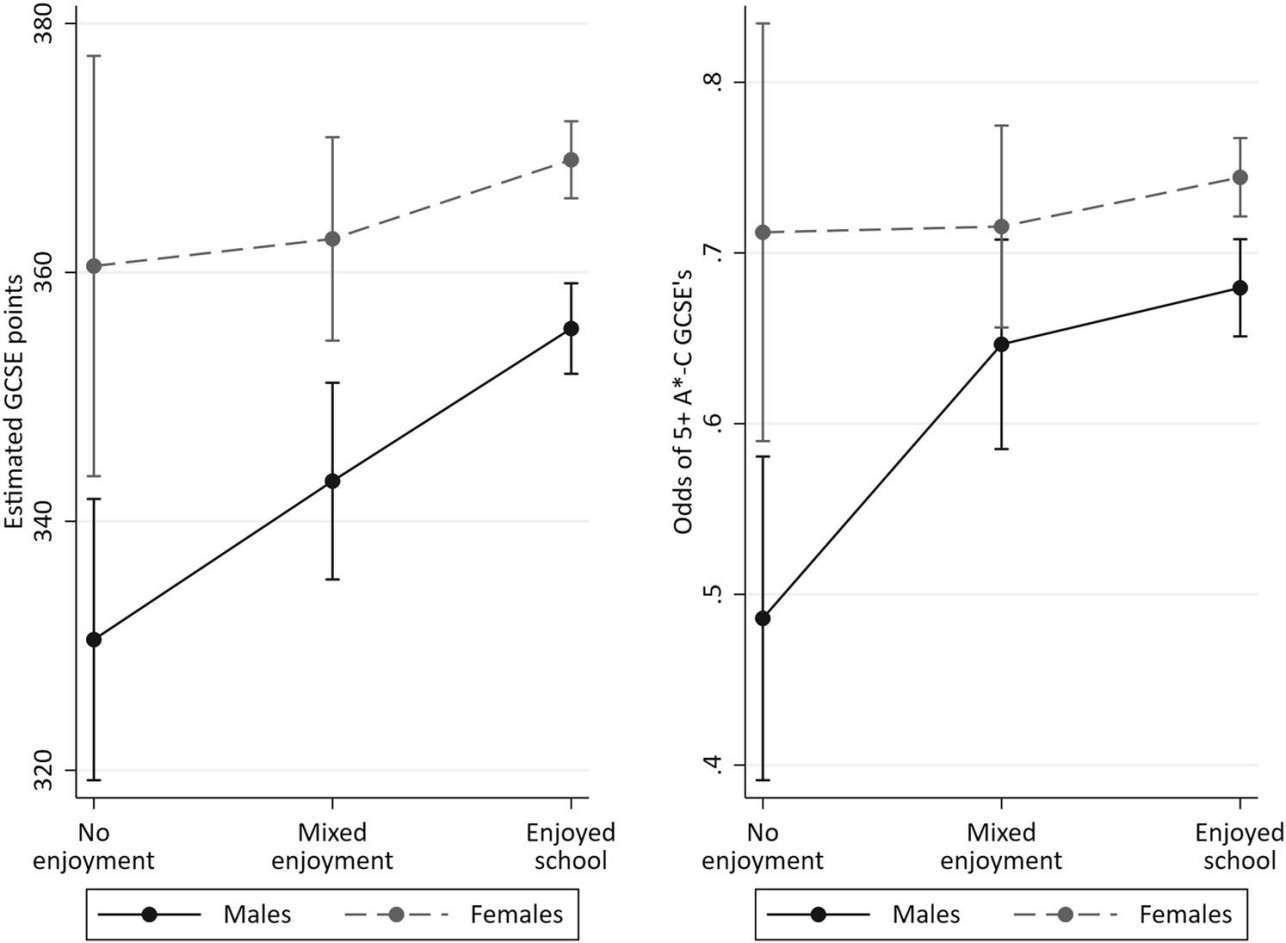
Interaction between school enjoyment and sex on GCSE achievement (capped points score). Left: linear regression of GCSE point scores. Right: odds ratios of attaining 5 + A*-C GCSE grades including Maths and English. GCSE General Certificate of Secondary Education. Bars represent confidence intervals.

There was little evidence of interactions in either the linear or logistic complete case analyses between school enjoyment and each of social class (interaction *p* values = 0.265 and 0.473 respectively), maternal education (*p* = 0.617; *p* = 0.591), cognitive ability (*p* = 0.867; *p* = 0.243) or pupil’s sex (*p* = 0.084; *p* = 0.174) on GCSE achievement. These interaction results should be interpreted with caution given the limited statistical power that exists to estimate them in the complete case sample. Figure 1 plots the sex-wise school enjoyment differences on achievement and demonstrates that while the gradient is steeper for males than females, there is insufficient precision in the estimates to draw firm conclusions of differences.

## Discussion

Our results highlight the potential importance of school enjoyment for educational achievement. Children in our imputed sample who enjoyed school at age 6 scored on average 29.34 more GCSE points at age 16 (equating to a half standard deviation increase (Table 1)) and were 64% more likely to attain 5 or more A*-C grades than children who did not enjoy school. Because the points score measure we used was a capped fine graded point score for GCSE’s at the time the cohort were in school, this represents a total difference of ~5 grades across the best eight GCSE’s. Given the importance of having 5 + A*-C GCSE grades to progress into further education and enter skilled jobs in the labour market^43^, lack of school enjoyment may provide an early indicator of pupils in need of more or better educational support. Our results corroborate those from previous studies on school enjoyment contemporaneously measured with achievement^13–15^ and extend this to exams sat 10 years after enjoyment was measured. That enjoying school is positively associated with achievement may be intuitive^11^, but it is remarkable that school enjoyment as early as age 6 explains differences in GCSE outcomes a decade later so well. The differences in achievement by enjoyment were almost as large as differences by parental occupational social class and sex, which have been widely acknowledged to be intervention worthy inequalities^39^. Furthermore, the binary indicators of school enjoyment that we used likely provided only a crude measure of the underlying liability or variation in school enjoyment amongst children. Better measurement of school enjoyment in future studies will help better understand the role that school enjoyment plays in educational achievement.

Associations of school enjoyment and achievement were not accounted for by other factors that are strongly related to education including socioeconomic position, cognitive ability and the home-learning environment^1,7,8,44^. There was attenuation in estimated effect sizes whereby children who enjoyed school at age 6 scored on average 14.41 more GCSE points at age 16 and were 29% more likely to attain 5 or more A*-C grades than children who did not enjoy school. These findings suggest that enjoyment of the learning environment reflects more than a propensity for innate cognitive ability or curiosity to learn^45,46^ and may capture something unique to educational outcomes. There is, of course, very high overlap between what cognitive ability tests seek to measure and what most GCSEs are designed to measure. Furthermore, the general lack of social patterning in enjoyment that we observed is surprising and suggests that children’s enjoyment does not just reflect social norms or parental behaviours that are patterned by socioeconomic position^41^. Associations between school enjoyment and achievement were also robust to other child level factors such as opinion of teachers, temperament, confidence in work and intelligence and quality of friendship groups. While these data do not allow us to provide causal evidence, our findings provide support for the notion that the relationship between school enjoyment and achievement may reflect a role of general school enjoyment in early childhood on later academic performance, rather than differences driven by other child level factors observed or not observed that commonly act as sources of confounding in educational research.

Because school enjoyment is potentially more modifiable than socioeconomic factors^11^ it potentially represents a promising intervention target for improving educational outcomes. Previous studies have demonstrated that school-based interventions can promote positive emotions such as enjoyment^24,25^ and may therefore offer long term opportunities for improving educational outcomes^10,26^. Positive emotional factors in learning environments has been found to decrease from childhood to adolescence^10,47^, so early schooling may represent the most effective period for interventions in terms of enabling long-term effects. These findings should not be taken in isolation as evidence that improving school enjoyment will improve educational outcomes or redress inequalities in education. Replication of this study’s findings in other settings and at other ages is required to inform when interventions are most likely to improve school enjoyment and how this may translate into better educational outcomes. However, for the purposes of identifying children who may need greater educational support, our results suggest that a simple measure of school enjoyment at an early age may have long-term predictive value to educators. One important implication from these findings is that some children gain little enjoyment from schooling. Due to the timeline of data collected, the ALSPAC data do not allow us to explore the reasons why some participants do not enjoy school. Lack of school enjoyment may arise from health problems or social, emotional and behavioural difficulties, so interventions on these upstream factors may be more beneficial and easier to implement than interventions on general school enjoyment. Further studies conducted on other data are required to investigate these reasons and explore how they may be best mitigated.

Strong sex differences existed whereby boys were more likely to dislike school than girls, conforming to previous research^35^. We found little evidence of interactions between school enjoyment and achievement by sex, but we had limited statistical power to investigate these. It is possible that school enjoyment plays a more important role in boys, than girls, education. Sex differences in education may reflect specific motivational differences^48^ or teacher biases^49^ which could lead to differential performance. Further work on larger samples is required to examine if sex differences exist in the relationship between enjoyment and achievement, to investigate the reasons that underlie sex differences in school enjoyment, and to determine how they could be reduced. Children who were younger in each school cohort were more likely to have lower achievement than those who were older, consistent with prior research into age and achievement^50,51^. However, we found little evidence that school enjoyment was patterned by age in year. There is continued debate about the impact of inflexible school starting ages used in some countries such as the UK^52^, and our results highlight that while children born later in the school year may perform less well in school, in our sample they do not derive any less enjoyment from schooling. Future work on samples from countries which have flexible school starting ages is required to see if these associations persist. From a statistical point of view, age in year may provide a valid instrument for instrumental variables analysis into the impacts of education.

This study has several limitations. First, the ALSPAC cohort does not provide a representative sample of the UK school population, meaning that our results may not be generalisable to the wider population of UK school children. Future research conducted on population representative samples is required for generalisability of these findings. That said, the associations that we investigated can be expected to hold in the wider population provided that participation in ALSPAC was not heavily stratified by school enjoyment and educational achievement^53^.

Second, while multiple imputation improved the sample size of our study and permitted inclusion of more participants than the complete case analysis, numerous assumptions are built into the data generating process that we used for the imputations. If important information were not included in the imputation process, then the imputation and subsequent results will be biased. Future research on datasets with a greater number of complete cases is required to demonstrate the replicability and external validity of these results. However, the results from the multiple imputation sample are likely to be less biased than those from our complete case sample because multiple imputation recovers participants and observation values that are excluded from complete case analyses. Where missingness is patterned with respect to variables included in the analyses, estimates from complete case samples may suffer from selection bias. Furthermore, our use of auxiliary variables in the imputation model will have accounted for underlying patterns in missingness that would not have been otherwise accounted for^42,54^.

Third, the associations here are estimated from observational data and therefore may arise due to further confounding factors between school enjoyment and educational achievement that we did not control for. Our results were robust to adjustment for some of the strongest drivers of educational achievement in the UK (family socioeconomic position, sex, ethnicity, age)^39^ so it is unlikely that unobserved confounding will induce sufficiently large bias to explain our results.

Fourth, because we condition our analyses on measures of cognitive ability, confidence in work and intelligence, and friendship groups that were collected at age 8, after the school enjoyment data, it is possible that this may have induced collider bias into our results^55^. Confounder variables should exert a downstream influence on exposure and outcome variables, and therefore adjusting for post-exposure variables can potentially introduce bias. Ability as measured by cognitive tests is highly stable over time^56^, meaning that our use of a cognitive ability measure obtained 2 years after school enjoyment is unlikely to induce sufficient collider bias to invalidate our results. Our intuition is that a child’s confidence in their work, confidence in their intelligence, and satisfaction with their friendship groups may vary over time in a manner that relates to school enjoyment. Given this, it is therefore possible that our results may be subject to collider bias induced by conditioning on downstream covariates. However, multiple imputation is expected to be less susceptible to this form of collider bias as analyses are not entirely conditioned on downstream data availability. The consistency in results between our complete case and multiple imputation analyse therefore provides confidence that our analyses are not strongly biased. There are no earlier measures of these variables in ALSPAC (or for cognitive ability no earlier measures with large enough sample size) to explore the potential impact of collider bias, but future research using datasets that have these variables and school enjoyment measured contemporaneously could explore this.

Fifth, we were unable to explicitly investigate school level effects of school enjoyment. The number of participants per school and class in ALSPAC varies considerably, reducing the statistical power to explore multilevel effects. Furthermore, the ALSPAC sample may not provide a representative cross-section of schools or classrooms, making it impossible to accurately distinguish school and classroom level variation. Future studies based upon representative samples of schools are required to assess school and class level determinants of school enjoyment.

Finally, our measure of how much the participants liked their teachers were reported by their mothers and therefore may not accurately reflect the child’s attitude to their teacher. Where a child’s school enjoyment is tied closely to the relationship with their teacher, reporting errors may lead to bias in our results.

In conclusion, our results suggest that school enjoyment at age 6 strongly associates with educational achievement at completion of compulsory schooling, and is not strongly patterned by family social background. Differences in achievement by school enjoyment are almost as large as socioeconomic and sex differences, and enjoyment-achievement associations are independent of these and other child level factors. Further observational and experimental studies into school enjoyment in other samples and schooling contexts are required to improve understanding of the drivers of school enjoyment and evaluate the potential of interventions to improve children’s enjoyment of schooling for educational achievement.

## Methods

### Study sample

Participants were children from ALSPAC. Pregnant women resident in Avon, UK with expected dates of delivery 1 April 1991 to 31 December 1992 were invited to take part in the study^57,58^. The initial number of pregnancies enrolled was 14,541, with 13,988 alive at the age of 1^57,58^. When the oldest children were ~7 years of age, the study was expanded to include eligible cases who had failed to join the study originally. This additional recruitment gave a total sample of 15,247 pregnancies and resulted in 14,899 children who were alive at 1 year of age^57,58^. For full details of the cohort profile and study design see^57,58^. The study website contains details of all the data that are available through a fully searchable data dictionary and variable search tool at http://www.bristol.ac.uk/alspac/researchers/our-data/. The initial ALSPAC sample was broadly representative of the UK population recorded in the 1991 Census. There was under representation of single parent families, those living in rented accommodation and some ethnic minorities^57,58^. Ethical approval for the study was obtained from the ALSPAC Ethics and Law Committee (ALEC) and the Local Research Ethics Committees. Informed consent for the use of data collected via questionnaires and direct assessments was obtained from participants following the recommendations of the ALEC. Informed consent was provided by the study mothers when the participants were minors. From the core sample of 14,899 children alive at one year, 3372 had full data. See Supplementary Fig. 1 for a STROBE diagram detailing the causes of attrition.

### Educational achievement

Our measure of educational achievement was fine graded point scores from age 16 examinations. The age 16 examinations represented the end of compulsory schooling at the time the ALSPAC cohort were in school. Fine graded point scores were used to obtain the richest measure of a child’s formal achievement currently available; there is greater variability than in level bandings or binary classifications such as achieving 5A*-C GCSEs, (the required standard for many further educational and occupational opportunities). Scores were obtained through data linkage to the UK National Pupil Database, which represents the most accurate record of individual educational achievement available in the UK. The database still does not cover all children in the UK: some private schools do not submit data to the NPD, and children who have been exclusively home schooled will also not have records.

### School enjoyment

At age 6 years (mean age 6.2 years, interquartile range 6.1–6.2) the ALSPAC participants were asked if they like school, with the response options of *yes* and *no*. Where participants selected both yes and no (*n* = 27) these responses were set to missing. At age 6.5 years (mean 6.5 years, IQR 6.5–6.6) they were asked how much they liked going to school, with the response options *I like it a lot*, *I like it a bit* and *I don’t like it*. Responses *I like it a lot* and *I like it a bit* were coded together to maintain consistency with the binary response at age 6 years. Our final school enjoyment variable was a three-category combination of these two responses, denoting (i) children who did not enjoy school at both occasions, (ii) children who enjoyed school at only one occasion and (iii) children who enjoyed school at both occasions. In the complete case analyses children with a missing value on either school enjoyment measure were excluded. Children in the UK start schooling in the September before their 5th birthday, so the ALSPAC cohort will have been in school for 1–2 years when school enjoyment was measured.

### Covariates

Our analysis adjusted for a range of covariates including sex; month of birth; ethnicity; cohort school year; the age at which children completed the enjoyment questionnaire; cognitive ability at age 8; highest maternal education and parental socioeconomic position. The ALSPAC cohort is split over three school years, so we use a three-category variable to indicate year and account for any temporal differences between the school year cohorts. Ethnicity was derived from mother reports and coded as white or non-white, to reflect the ethnic homogeneity of the cohort. Mothers highest level of education was obtained during pregnancy, with the responses of Degree, A-level, O-level, vocational or certificate of secondary education (CSE). We combined vocational and CSE due to low responses. Parental socioeconomic position was measured as occupational social class from mothers reports of her own and the fathers, occupations. The highest two (professional occupations and managerial and technical occupations) and the lower two categories (partly skilled occupations and unskilled occupations) were respectively combined due to low numbers. Armed forces personnel were set to missing due to the inability to distinguish seniority. In dual parent households, the highest of the two parents’ occupational social class was used. In single parent households the mothers, occupational social class was used (note that ALSPAC principally followed the mothers). Childrens, cognitive ability was assessed using the short form of the Wechsler Intelligence Scale for Children (WISC)^59^ at an ALSPAC direct assessment visit when the children were aged 8. The WISC was the most widely validated cognitive ability scale at the time of measurement, with the short form version having high reliability^60^. We use reports by the mothers regarding how much their children like their teachers at age 6, based on a four-point Likert scale. Child temperament was measured using a score built from seven self-report responses to questions relating to different aspects of the childs, temperament at age 6 such as how happy they are and how frequently they are angry. Childrens, confidence in their work quality and intelligence were measured from self-reports at age 8. Confidence in work quality was measured from four responses on how interesting they found their schoolwork, their opinion of the quality of their work, how well they compete the work, and their perception of their teacher’s opinion of their work. Confidence in their intelligence measured from three responses to how hard or easy they found their work and how clever they believed themselves to be. We use a measure of the family home learning environment combined from parent responses to six questions about the learning environment that they provide for their child at age 3^44^. These questions respond to the frequency that they teach their child colours, the alphabet, numbers, nursery rhymes, songs, and shapes and sizes^44^. Finally, we used a measure of the quality of a childs, friendship group at age 8 based upon five self-report responses on how happy the child was with their number of friends, whether they often see their friends outside of school, whether their friends understand them, whether they talk to their friends about their problems, and how happy they are with their friends overall.

### Multiple imputation

Given differential patterns of missingness amongst variables and longitudinal cohort attrition leading to restricted numbers for complete case analysis after listwise deletion, we conducted Multiple Imputation by Chained Equations (MICE)^61^ to impute missing data. We imputed data on all variables except for sex, month of birth and ethnicity, resulting in an imputed sample size of 12,135. From these 12,135 children, only 2544 (20.96%) had full data on outcomes, exposures and all covariates (Supplementary Fig. 1). However, 82% had a measure of achievement and 63% had a measure of school enjoyment. Item missingness increased in a broadly linear manner (Supplementary Fig. 2). While the proportion of missing data is large for some variables, previous work has demonstrated that this will not bias imputation results^54^. Indeed, the proportion of missing data in the complete-case sample should not be used to provide a guide on the comparative accuracy of analyses on multiply imputed or complete case data^54^. Multiple imputation assumes that the values are missing at random, conditional upon the data included in the imputation model. If this assumption is violated, then results may be biased. To help overcome this problem and to increase the accuracy and reliability of the multiply imputed data, we make use of additional auxiliary variables in the imputation model as indicated in Supplementary Table 1. The use of auxiliary variables increases the ability of multiple imputation models to account for underlying patterns in missingness that would be ignored and therefore bias the complete-case analysis^42,54^. Given that the likelihood that a participant was a complete case in our dataset is likely to remain correlated to our outcome variable even after consideration of all covariates, it is expected that a complete case sample is a highly selected subsample and estimates based can be more biased than a multiple imputation analysis^42^. Hughes et al.^42^ demonstrate that in almost all scenarios multiple imputation outperforms complete case analysis in terms of robustness to bias, particularly where there exists patterns of differential attrition and non-response. Unlike complete case analysis, multiple imputation can recover participants and observation values that would otherwise be excluded. Where missingness is patterned with respect to particular participants or observation values (such as those from lower socioeconomic position households^57^), multiple imputation will provide results that are more balanced and generalisable to the wider cohort than complete case analysis. 100 datasets were imputed for the analyses. The supplementary information file contains further details on the imputation process.

### Statistical analysis

We estimated the odds of mixed enjoyment or enjoying school compared to not enjoying school using multinomial logistic regression. We estimated the association of school enjoyment and educational achievement using linear regression for fine graded GCSE point scores and logistic regression for obtaining 5 + A*-C GCSEs. Three sets of models are used for each modelling approach; the first is an unadjusted analysis controlling only for sex of child, month of birth, ethnicity, cohort school year and age at enjoyment questionnaire completion; the second additionally controls for parental occupational social class and mothers highest education; the third additionally controls for the child’s cognitive ability. We present results here for the multiple imputed datasets; results from the complete case analyses are presented in the supplementary tables.

### Reporting summary

Further information on research design is available in the Nature Research Reporting Summary linked to this article.

## Supplementary information

Supplementary information

Reporting Summary

## Data Availability

The data that support the findings of this study are available from the ALSPAC study, but restrictions apply to the availability of these data, which were used under license for the current study, and so are not publicly available. The data are available subject to approval from the ALSPAC study Executive. The empirical dataset used in this study has been archived with the ALSPAC study under the project identifier B2193. For further information please see http://www.bristol.ac.uk/alspac/researchers/.

## Code availability

All code used to process and analyse the data are available at https://github.com/timtmorris/ALSPAC_school_enjoyment.
