# Supplementary material for: Associations between school enjoyment at age 6 and later educational achievement: evidence from a UK cohort study
Source: NPJ Sci Learn. 2021 Jun 15;6:18. doi: 10.1038/s41539-021-00092-w (PMC8206254; doi:10.1038/s41539-021-00092-w)
Supplement: Supplementary file 1 — Supplementary information [file 41539_2021_92_MOESM1_ESM.pdf]

Supplementary Information to:

Associations between school enjoyment at age 6 and later educational achievement: evidence from a UK cohort study.

Tim T Morris * [1,2], Danny Dorling [3], Neil M Davies [1,2,4], George Davey Smith [1,2].

1. MRC Integrative Epidemiology Unit at the University of Bristol, BS8 2BN, United Kingdom.
2. Population Health Sciences, Bristol Medical School, University of Bristol, Barley House, Oakfield Grove, Bristol, BS8 2BN, United Kingdom.
3. School of Geography and the Environment, University of Oxford, Oxford, OX1 3QY, United Kingdom.
4. K.G. Jebsen Center for Genetic Epidemiology, Department of Public Health and Nursing, NTNU, Norwegian University of Science and Technology, Norway.

Multiple imputation

Multiple Imputation by Chained Equations (MICE) (Royston & White, 2011) was conducted to impute missing data. From the 14,899 children who were alive at one year of age only 2,544 had full data on outcomes, exposures and all covariates (Figure S1). Data were imputed for all variables except for sex, month of birth and ethnicity, resulting in an imputed sample size of 12,135. We imputed 100 datasets for analyses. Table S1 displays the extent of missingness in the cohort. Due to patterns of missingness across the variables of interest, only 20.96% of ALSPAC participants with sex, month of birth and ethnicity data have full data on all other variables, with item missingness increasing in a broadly linear manner. The numbers of responses for each variable prior to imputation and the imputation regressions used to impute the variables are displayed in Figure S2.

Supplementary Figure 1: STROBE diagram of attrition in the complete case sample.

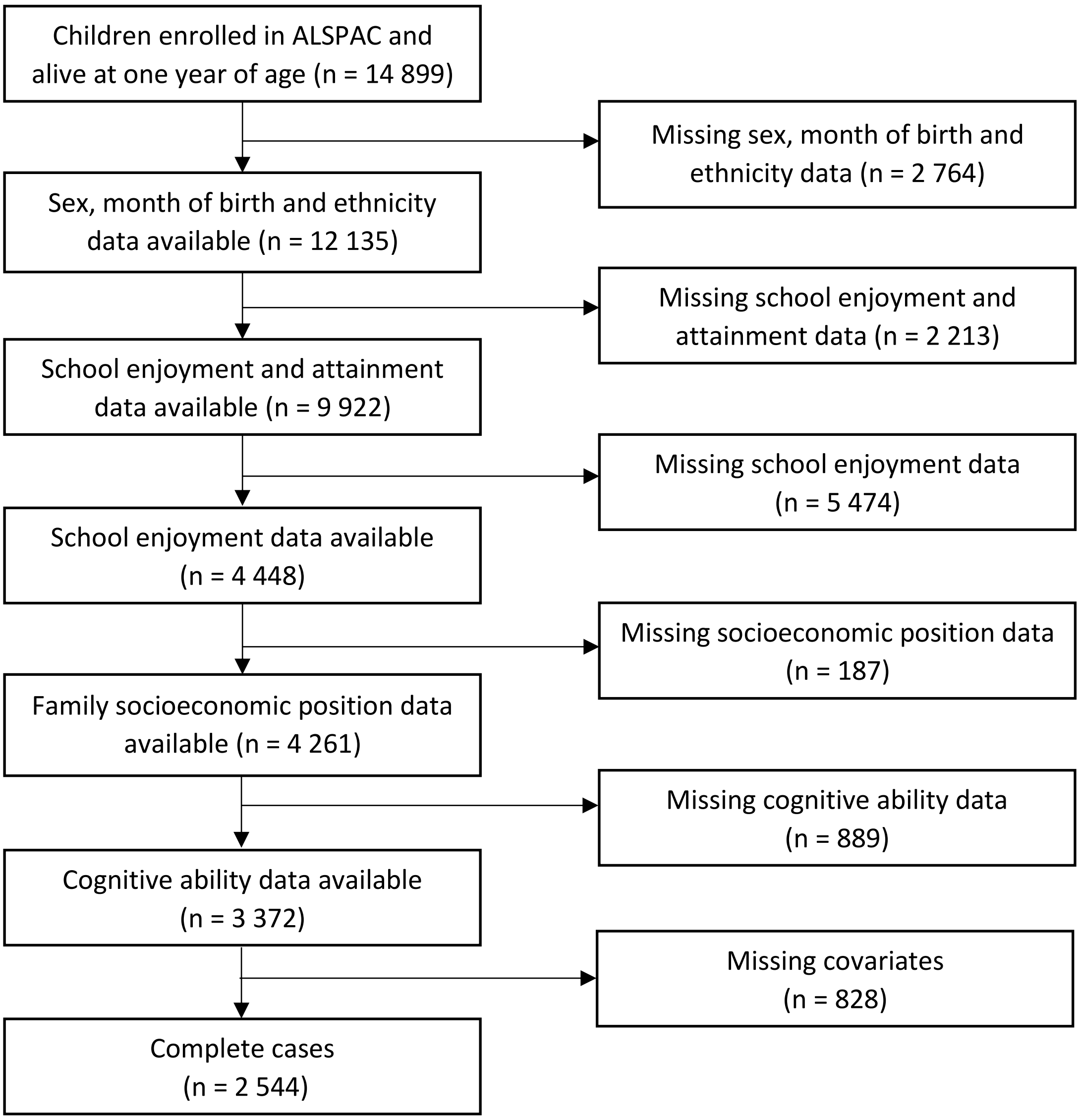

Supplementary Figure 2: Number of missing items in the ALSPAC sample from the 12,135 participants who were alive at one year of age and had data on biological sex at birth, month of birth and ethnicity.

Number of missing items	n	%
0	2,544	20.96
1	916	7.55
2	1,351	11.13
3	1,137	9.37
4	702	5.78
5	864	7.12
6	678	5.59
7	722	5.95
8	883	7.28
9	1,320	10.88
10	348	2.87
11	248	2.04
12	368	3.03
13	53	0.44
14	2	0.02

Supplementary Table 1: Regression commands used in multiple imputation. GCSE: General Certificate of Secondary Education.

Variable	Response N	Regression command	Prediction equation
Female	12,135		[No missing data in estimation sample].
Month of birth	12,135		[No missing data in estimation sample].
Ethnicity	12,135		[No missing data in estimation sample].
Maternal education	12,068	Ordered logistic	Enjoyment age 6; Enjoyment age 6.5; GCSE 5+ A*-C; GCSE points; Female; Month of birth; Ethnicity; Social class; Cognitive ability; School year.
Social class	11,320	Ordered logistic	Enjoyment age 6; Enjoyment age 6.5; GCSE 5+ A*-C; GCSE points; Female; Month of birth; Ethnicity; Maternal education; Cognitive ability; School year.
School year	10,514	Multinomial logistic	Age in weeks at return of age 6 questionnaire; Recieved date of age 6 questionnaire; Age in weeks at return of age 6.5 questionnaire; Recieved date of age 6.5 questionnaire.
GCSE 5+ A*-C	10,009	Logistic	Enjoyment age 6; Enjoyment age 6.5; GCSE points; Female; Month of birth; Ethnicity; Maternal education; Social class; Cognitive ability; School year; Child's opinion of teacher; Child's temperament; Child's confidence in work quality; Child's confidence in their intelligence; Home learning environment; Friends score; Child's personality on "Big 5" personality scales.
GCSE points	10,009	Linear	Enjoyment age 6; Enjoyment age 6.5; GCSE 5+ A*-C; Female; Month of birth; Ethnicity; Maternal education; Social class; Cognitive ability; School year; Child's opinion of teacher; Child's temperament; Child's confidence in work quality; Child's confidence in their intelligence; Home learning environment; Friends score; Child's personality on "Big 5" personality scales.
Enjoyment at age 6	6,856	Logistic	Enjoyment age 6; Enjoyment age 6.5; GCSE 5+ A*-C; GCSE points; Female; Month of birth; Ethnicity; Maternal education; Social class; Cognitive ability; School year; Child's opinion of teacher; Child's temperament; Child's confidence in work quality; Child's confidence in their intelligence; Home learning environment; Friends score; Child's personality on "Big 5" personality scales.
Age at age 6 questionnaire	6,991	Linear	Enjoyment age 6; Enjoyment age 6.5; GCSE 5+ A*-C; GCSE points; Month of birth; School year; Age in weeks at return of age 6 questionnaire.
Enjoyment at age 6.5	5,246	Logistic	Enjoyment age 6; Enjoyment age 6.5; GCSE 5+ A*-C; GCSE points; Female; Month of birth; Ethnicity; Maternal education; Social class; Cognitive ability; School year; Child's opinion of teacher; Child's temperament; Child's confidence in work quality; Child's confidence in their intelligence; Home learning environment; Friends score; Child's personality on "Big 5" personality scales.
Age at age 6.5 questionnaire	6,109	Linear	Enjoyment age 6; Enjoyment age 6.5; GCSE 5+ A*-C; GCSE points; Month of birth; School year; Age in weeks at return of age 6.5 questionnaire; Child's self identication of height most similar to them at age 6.5; Child's self identication of weight most similar to them at age 6.5.
Cognitive ability	6,682	Linear	Enjoyment age 6; Enjoyment age 6.5; GCSE 5+ A*-C; GCSE points; Female; Month of birth; Ethnicity; Maternal education; Social class; School year.

Home learning environment (individual components)	9,487	Linear	Enjoyment age 6; Enjoyment age 6.5; GCSE points; Female; Month of birth; Ethnicity; Maternal education; Social class; Cognitive ability; School year; Child's opinion of teacher; Child's confidence in work quality; Child's confidence in their intelligence; Child's personality on "Big 5" personality scales.
Child's opinion of teacher	8,027	Ordered logistic	Enjoyment age 6; Enjoyment age 6.5; GCSE points; Female; Month of birth; Ethnicity; Maternal education; Social class; Cognitive ability; School year; Child's temperament; Child's confidence in work quality; Child's confidence in their intelligence; Home learning environment; Friends score; Child's personality on "Big 5" personality scales; Child's opinion of teacher at age 7; Child's opinion of teacher at age 8.
Child's temperament (individual components)	5,939	Ordered logistic	Enjoyment age 6; Enjoyment age 6.5; GCSE points; Female; Month of birth; Ethnicity; Maternal education; Social class; Cognitive ability; School year; Child's opinion of teacher; Child's confidence in work quality; Child's confidence in their intelligence; Home learning environment; Friends score; Child's personality on "Big 5" personality scales.
Child's cofidence in work quality (individual components)	7,053	Ordered logistic	Enjoyment age 6; Enjoyment age 6.5; GCSE points; Female; Month of birth; Ethnicity; Maternal education; Social class; Cognitive ability; School year; Child's temperament; Child's opinion of teacher; Child's confidence in their intelligence; Home learning environment; Friends score; Child's personality on "Big 5" personality scales.
Child's cofidence in their intelligence (individual components)	7,051	Ordered logistic	Enjoyment age 6; Enjoyment age 6.5; GCSE points; Female; Month of birth; Ethnicity; Maternal education; Social class; Cognitive ability; School year; Child's temperament; Child's opinion of teacher; Child's confidence in work quality; Home learning environment; Friends score; Child's personality on "Big 5" personality scales.
Friends score (individual components)	6,356	Ordered logistic	Enjoyment age 6; Enjoyment age 6.5; GCSE points; Female; Month of birth; Ethnicity; Maternal education; Social class; Cognitive ability; School year; Child's temperament; Child's opinion of teacher; Child's confidence in work quality; Child's confidence in work quality; Home learning environment; Child's personality on "Big 5" personality scales.

Supplementary Table 2: Multinomial regression of school enjoyment in multiple imputation sample. GCSE: General Certificate of Secondary Education; CI: confidence interval; Ref: reference category; CSE: Certificate of Secondary Education; Q1: First enjoyment questionnaire at age 6; Q2: Second enjoyment questionnaire at age 6.5.

	Model 1: Unadjusted			Model 2: Family socioeconomic position			Model 3: IQ adjusted			Model 4: Fully adjusted		
	OR	95% CI	p value	OR	95% CI	p value	OR	95% CI	p value	OR	95% CI	p value
No enjoyment	*Ref*			*Ref*			*Ref*			*Ref*		
Mixed enjoyment												
Social class												
IV & V	*Ref*									*Ref*		
III Manual	0.93	0.69, 1.26	0.633							0.89	0.65, 1.21	0.45
III Non-manual	1.01	0.72, 1.41	0.954							0.93	0.66, 1.32	0.692
I & II	0.98	0.72, 1.35	0.919							0.87	0.61, 1.25	0.463
Maternal education												
CSE/vocational				*Ref*						*Ref*		
O-level				1.12	0.86, 1.47	0.397				1.12	0.86, 1.47	0.397
A-level				0.98	0.73, 1.31	0.877				0.98	0.73, 1.31	0.877
Degree				1.30	0.91, 1.85	0.145				1.30	0.91, 1.85	0.145
Cognitive ability							1.08	0.97, 1.2	0.141	1.03	0.91, 1.17	0.604
Female	1.7	1.36, 2.13	<0.001	1.70	1.36, 2.13	<0.001	1.70	1.36, 2.13	<0.001	1.48	1.17, 1.86	0.001
Month of birth	0.98	0.94, 1.01	0.199	0.98	0.94, 1.01	0.196	0.98	0.94, 1.01	0.186	0.99	0.96, 1.03	0.648
Non-white	1.26	0.69, 2.29	0.448	1.27	0.7, 2.3	0.437	1.27	0.7, 2.32	0.431	1.40	0.76, 2.58	0.285
School year												
2006/2007	*Ref*			*Ref*			*Ref*			*Ref*		
2007/2008	0.99	0.74, 1.31	0.93	0.99	0.74, 1.31	0.932	0.99	0.74, 1.32	0.941	0.99	0.74, 1.33	0.971
2008/2009	0.94	0.62, 1.43	0.788	0.94	0.62, 1.43	0.788	0.95	0.63, 1.44	0.814	0.97	0.63, 1.48	0.883
Age at Q1	0.67	0.34, 1.29	0.23	0.66	0.34, 1.29	0.228	0.67	0.34, 1.31	0.242	0.71	0.36, 1.39	0.315
Age at Q2	0.86	0.4, 1.82	0.689	0.86	0.4, 1.82	0.686	0.86	0.41, 1.84	0.703	0.87	0.4, 1.93	0.737
Home learning environment										0.99	0.91, 1.08	0.765

Likes teacher												
Not at all										*Ref*		
Sometimes										1.09	0.5, 2.4	0.825
Usually										1.55	0.77, 3.12	0.224
Always										2.34	1.14, 4.8	0.021
Temperament										1.08	1.02, 1.14	0.011
Work confidence										1.16	1.09, 1.23	<0.001
Intelligence confidence										1.00	0.93, 1.07	0.978
Friends score										0.97	0.92, 1.02	0.18
Enjoyed school												
Social class												
IV & V	*Ref*									*Ref*		
III Manual	0.99	0.76, 1.29	0.941							0.90	0.67, 1.19	0.45
III Non-manual	1.16	0.87, 1.55	0.305							0.98	0.71, 1.34	0.886
I & II	1.09	0.82, 1.47	0.55							0.87	0.61, 1.24	0.443
Maternal education												
CSE/vocational				Ref						*Ref*		
O-level				1.15	0.91, 1.46	0.251				1.09	0.84, 1.41	0.526
A-level				1.12	0.86, 1.45	0.411				1.04	0.77, 1.41	0.778
Degree				1.36	0.99, 1.87	0.06				1.33	0.9, 1.96	0.156
Cognitive ability							1.24	1.11, 1.38	<0.001	1.16	1.02, 1.32	0.025
Female	2.61	2.1, 3.24	<0.001	2.61	2.1, 3.24	<0.001	2.62	2.1, 3.25	<0.001	1.97	1.56, 2.48	<0.001
Month of birth	0.95	0.92, 0.99	0.005	0.95	0.92, 0.98	0.004	0.95	0.92, 0.98	0.003	0.98	0.94, 1.01	0.213
Non-white	1.52	0.85, 2.74	0.159	1.53	0.85, 2.75	0.157	1.56	0.86, 2.8	0.142	1.87	0.99, 3.55	0.056
School year												
2006/2007	*Ref*			*Ref*			*Ref*			*Ref*		
2007/2008	0.91	0.7, 1.18	0.488	0.91	0.71, 1.18	0.497	0.92	0.71, 1.19	0.525	0.93	0.71, 1.23	0.62
2008/2009	0.82	0.56, 1.22	0.331	0.83	0.56, 1.22	0.338	0.84	0.57, 1.24	0.382	0.87	0.57, 1.32	0.5

Age at Q1	0.69	0.38, 1.25	0.222	0.68	0.38, 1.24	0.214	0.71	0.39, 1.29	0.263	0.75	0.4, 1.42	0.375
Age at Q2	0.7	0.34, 1.42	0.325	0.70	0.34, 1.42	0.317	0.71	0.35, 1.45	0.352	0.73	0.34, 1.56	0.414
Home learning environment										1.00	0.92, 1.08	0.955
Likes teacher												
Not at all										*Ref*		
Sometimes										1.61	0.74, 3.5	0.231
Usually										3.60	1.75, 7.4	0.001
Always										9.44	4.45, 20.02	<0.001
Temperament										1.19	1.12, 1.26	<0.001
Work confidence										1.36	1.27, 1.45	<0.001
Intelligence confidence										0.97	0.91, 1.04	0.423
Friends score										0.94	0.9, 0.99	0.017

Supplementary Table 3: Multinomial regression of school enjoyment in complete case sample. OR: Odds Ratio; CI: confidence interval; Ref: reference category; CSE: Certificate of Secondary Education; Q1: First enjoyment questionnaire at age 6; Q2: Second enjoyment questionnaire at age 6.5.

	Model 1: Occupational social class			Model 2: Maternal education			Model 3: Intelligence			Model 4: Fully adjusted		
	OR	95% CI	p value	OR	95% CI	p value	OR	95% CI	p value	OR	95% CI	p value
**No enjoyment**	Ref			Ref			Ref			Ref		
**Mixed enjoyment**												
Social class												
IV & V	*Ref*									*Ref*		
III Manual	0.93	0.5, 1.71	0.804							0.88	0.46, 1.66	0.688
III Non-manual	1.3	0.68, 2.47	0.426							1.41	0.72, 2.76	0.313
I & II	1.27	0.69, 2.33	0.451							1.36	0.67, 2.75	0.397
Maternal education												
CSE/vocational				*Ref*						*Ref*		
O-level				0.86	0.48, 1.56	0.62				0.87	0.47, 1.61	0.66
A-level				0.7	0.38, 1.3	0.263				0.64	0.33, 1.24	0.183
Degree				1.04	0.51, 2.13	0.91				0.92	0.4, 2.15	0.855
Cognitive ability							0.98	0.79, 1.22	0.863	0.93	0.73, 1.18	0.547
Female	2.13	1.37, 3.31	0.001	2.1	1.35, 3.26	0.001	2.1	1.36, 3.26	0.001	2.02	1.29, 3.17	0.002
Month of birth	1.01	0.93, 1.08	0.883	1.01	0.94, 1.08	0.832	1.01	0.94, 1.08	0.837	1.02	0.95, 1.1	0.634
Non-white	1.53	0.32, 7.23	0.589	1.6	0.34, 7.52	0.554	1.58	0.34, 7.44	0.563	1.63	0.34, 7.78	0.54
School year												
2006/2007	*Ref*			*Ref*			*Ref*			*Ref*		
2007/2008	1.04	0.59, 1.84	0.894	1.07	0.61, 1.89	0.817	1.07	0.61, 1.89	0.813	1.1	0.61, 1.97	0.751
2008/2009	1.3	0.53, 3.17	0.568	1.32	0.54, 3.21	0.545	1.33	0.55, 3.24	0.526	1.46	0.58, 3.64	0.418
Age at Q1	0.17	0.03, 1.11	0.064	0.17	0.03, 1.09	0.061	0.17	0.03, 1.06	0.057	0.24	0.04, 1.48	0.123
Age at Q2	0.95	0.13, 6.8	0.963	0.92	0.14, 6.17	0.93	0.96	0.14, 6.54	0.966	0.85	0.12, 5.95	0.869
Home learning environment										1	0.83, 1.21	0.981
Likes teacher												
Not at all										*Ref*		

Sometimes										1.27	0.18, 9	0.814
Usually										1.35	0.2, 8.88	0.758
Always										1.85	0.28, 12.28	0.526
Temperament										0.97	0.88, 1.07	0.571
Work confidence										1.1	0.99, 1.23	0.077
Intelligence confidence										1.08	0.92, 1.27	0.33
Friends score										0.94	0.86, 1.02	0.139
Constant	1.55	0.59, 4.05	0.373	1.94	0.73, 5.16	0.184	1.67	0.7, 4.03	0.25	0.35	0.01, 8.43	0.52
**Enjoyed school**												
Social class												
IV & V	*Ref*									*Ref*		
III Manual	1.01	0.59, 1.73	0.973							0.77	0.44, 1.37	0.382
III Non-manual	1.73	0.98, 3.08	0.06							1.52	0.82, 2.81	0.179
I & II	1.54	0.89, 2.66	0.119							1.17	0.61, 2.21	0.641
Maternal education												
CSE/vocational				*Ref*								
O-level				1.09	0.64, 1.86	0.747				1.01	0.57, 1.78	0.969
A-level				1.08	0.63, 1.86	0.784				0.87	0.47, 1.59	0.645
Degree				1.41	0.74, 2.69	0.299				1.16	0.53, 2.51	0.712
Cognitive ability							1.23	1.02, 1.49	0.033	1.12	0.9, 1.4	0.295
Female	3.1	2.09, 4.6	<0.001	3.05	2.06, 4.52	<0.001	3.08	2.08, 4.58	<0.001	2.46	1.63, 3.71	<0.001
Month of birth	0.96	0.9, 1.02	0.164	0.96	0.9, 1.02	0.19	0.96	0.9, 1.02	0.172	0.97	0.91, 1.04	0.349
Non-white	1.99	0.48, 8.3	0.344	2.07	0.5, 8.6	0.318	2.01	0.48, 8.35	0.338	2.17	0.5, 9.34	0.299
School year												
2006/2007	*Ref*			*Ref*			*Ref*			*Ref*		
2007/2008	0.95	0.57, 1.58	0.849	0.99	0.6, 1.64	0.973	0.99	0.6, 1.64	0.961	1.05	0.62, 1.78	0.862
2008/2009	0.93	0.42, 2.07	0.861	0.95	0.43, 2.11	0.907	0.97	0.44, 2.15	0.948	1.15	0.5, 2.65	0.74
Age at Q1	0.58	0.14, 2.33	0.44	0.55	0.14, 2.16	0.39	0.57	0.15, 2.26	0.428	0.89	0.21, 3.85	0.88

Age at Q2	0.97	0.17, 5.44	0.973	0.95	0.18, 4.97	0.947	0.96	0.18, 5.14	0.959	0.77	0.14, 4.35	0.77
Home learning environment										0.99	0.84, 1.18	0.949
Likes teacher												
Not at all										*Ref*		
Sometimes										0.86	0.13, 5.49	0.872
Usually										2.2	0.37, 13.01	0.386
Always										6.33	1.06, 37.73	0.043
Temperament										1.12	1.02, 1.23	0.022
Work confidence										1.29	1.17, 1.43	<0.001
Intelligence confidence										0.98	0.85, 1.14	0.811
Friends score										0.92	0.85, 1	0.048
Constant	9.95	4.26, 23.21	<0.001	11.22	4.71, 26.72	<0.001	12.27	5.66, 26.63	<0.001	0.03	0, 0.58	0.02

Supplementary Table 4: Linear regression of GCSE points at age 16 in multiple imputation sample. OR: Odds Ratio; CI: confidence interval; Ref: reference category; CSE: Certificate of Secondary Education; Q1: First enjoyment questionnaire at age 6; Q2: Second enjoyment questionnaire at age 6.5.

	Model 1: Unadjusted			Model 2: Family socioeconomic position			Model 3: IQ adjusted		
	Beta	95% CI	p value	Beta	95% CI	p value	Beta	95% CI	p value
School enjoyment									
No enjoyment	Ref			Ref			Ref		
Mixed enjoyment	12.53	2.42, 22.65	0.015	8.05	0.22, 15.89	0.044	6.16	-1.7, 14.02	0.125
Enjoyed school	29.34	19.4, 39.29	<0.001	17.75	10.45, 25.05	<0.001	14.41	6.9, 21.93	<0.001
Social class									
IV & V				Ref			Ref		
III Manual				6.75	2.78, 10.71	0.001	6.25	2.31, 10.19	0.002
III Non-manual				23.03	18.76, 27.3	<0.001	21.72	17.5, 25.95	<0.001
I & II				23.24	18.5, 27.97	<0.001	22.19	17.49, 26.89	<0.001
Maternal education									
CSE/vocational				Ref			Ref		
O-level				20.64	17.14, 24.13	<0.001	19.37	15.91, 22.84	<0.001
A-level				33.40	29.14, 37.66	<0.001	31.26	27.02, 35.5	<0.001
Degree				45.80	40.25, 51.35	<0.001	43.84	38.37, 49.31	<0.001
Cognitive ability				44.37	42.63, 46.11	<0.001	43.01	41.25, 44.77	<0.001
Female	21.99	18.61, 25.37	<0.001	22.73	19.88, 25.59	<0.001	21.61	18.73, 24.48	<0.001
Month of birth	-1.19	-1.75, -0.62	<0.001	-1.60	-2.07, -1.12	<0.001	-1.47	-1.95, -0.99	<0.001
Non-white	-7.61	-15.48, 0.27	0.058	-3.03	-10.02, 3.97	0.396	-1.96	-8.86, 4.94	0.577
School year									
2006/2007	Ref			Ref			Ref		
2007/2008	4.81	0.06, 9.57	0.047	5.47	1.72, 9.23	0.004	5.03	1.27, 8.8	0.009
2008/2009	9.89	2.86, 16.92	0.006	11.54	5.88, 17.2	<0.001	10.56	4.88, 16.23	<0.001
Age at Q1	-17.18	-30.82, -3.55	0.014	-8.84	-19.08, 1.4	0.09	-7.16	-17.39, 3.07	0.17
Age at Q2	-14.67	-33.98, 4.64	0.136	-8.56	-21.99, 4.86	0.211	-7.25	-20.63, 6.14	0.289
Home learning environment							3.34	2.06, 4.61	<0.001

Likes teacher									
Not at all							Ref		
Sometimes							9.24	-14.54, 33.02	0.446
Usually							15.79	-6.43, 38.02	0.164
Always							17.73	-4.62, 40.08	0.12
Temperament							0.75	-0.27, 1.77	0.148
Work confidence							-0.47	-1.68, 0.73	0.442
Intelligence confidence							3.62	2.65, 4.6	<0.001
Friends score							-0.02	-0.8, 0.77	0.967
Constant	294.27	282.89, 305.64	<0.001	276.35	267.03, 285.67	<0.001	206.58	174.45, 238.7	<0.001

Supplementary Table 5: Linear regression of GCSE points at age 16 in complete case sample. GCSE: General Certificate of Secondary Education; CI: confidence interval; Ref: reference category; CSE: Certificate of Secondary Education; Q1: First enjoyment questionnaire at age 6; Q2: Second enjoyment questionnaire at age 6.5.

	Model 1: Unadjusted			Model 2: Family socioeconomic position			Model 3: IQ adjusted		
	Beta	95% CI	p value	Beta	95% CI	p value	Beta	95% CI	p value
School enjoyment									
No enjoyment	Ref			Ref			Ref		
Mixed enjoyment	11.25	-2.71, 25.21	0.114	11.17	0.29, 22.05	0.044	14.65	4.25, 25.05	0.006
Enjoyed school	31.41	19.03, 43.8	<0.001	21.88	12.22, 31.55	<0.001	25.18	15.81, 34.56	<0.001
Social class									
IV & V				Ref			Ref		
III Manual				9.8	2.99, 16.61	0.005	6.65	0, 13.31	0.05
III Non-manual				21.32	14.6, 28.05	<0.001	20.39	13.79, 26.98	<0.001
I & II				22.52	15.35, 29.7	<0.001	21.38	14.34, 28.42	<0.001
Maternal education									
CSE/vocational				Ref			Ref		
O-level				15.18	8.88, 21.48	<0.001	15.46	9.35, 21.58	<0.001
A-level				26.86	20.07, 33.65	<0.001	27.11	20.44, 33.77	<0.001
Degree				33.44	25.2, 41.69	<0.001	33.61	25.45, 41.76	<0.001
Cognitive ability				35.46	33.03, 37.88	<0.001	34.85	32.43, 37.27	<0.001
Female	13.89	8.4, 19.39	<0.001	18.67	14.38, 22.96	<0.001	18.66	14.37, 22.94	<0.001
Month of birth	-0.52	-1.47, 0.43	0.283	-1.13	-1.87, -0.39	0.003	-1.09	-1.82, -0.37	0.003
Non-white	-0.41	-15.54, 14.71	0.957	-6.69	-18.49, 5.11	0.266	-5.68	-17.3, 5.94	0.338
School year									
2006/2007	Ref			Ref			Ref		
2007/2008	8.74	0.93, 16.55	0.028	6.11	0.02, 12.2	0.049	7.19	1.26, 13.12	0.017
2008/2009	11.89	0.44, 23.33	0.042	10.13	1.2, 19.05	0.026	11.73	2.97, 20.5	0.009
Age at Q1	-20.2	-45.38, 4.98	0.116	-11.17	-30.83, 8.49	0.265	-4.21	-23.11, 14.69	0.663
Age at Q2	8.69	-15.6, 32.97	0.483	-1.82	-20.75, 17.11	0.85	-5.62	-24.11, 12.87	0.551
Home learning environment							3.36	1.34, 5.39	0.001
Likes teacher									
Not at all							Ref		
Sometimes							1.9	-30.05, 33.85	0.907

Usually							12.28	-18.25, 42.8	0.43
Always							13.18	-17.3, 43.65	0.397
Temperament							0.26	-0.86, 1.37	0.653
Work confidence							0.47	-0.64, 1.59	0.403
Intelligence confidence							0.39	-1.19, 1.97	0.627
Friends score							0.13	-0.78, 1.04	0.783
Constant	321.51	305.09, 337.93	<0.001	291.31	277.18, 305.44	<0.001	237.79	198.04, 277.54	<0.001

Supplementary Table 6: Logistic regression of GCSE points at age 16 in multiple imputation sample. OR: Odds Ratio; CI: confidence interval; GCSE: General Certificate of Secondary Education; CI: confidence interval; Ref: reference category; CSE: Certificate of Secondary Education; Q1: First enjoyment questionnaire at age 6; Q2: Second enjoyment questionnaire at age 6.5.

	Model 1: Unadjusted			Model 2: Family socioeconomic position			Model 3: IQ adjusted		
	OR	95% CI	p value	OR	95% CI	p value	OR	95% CI	p value
School enjoyment									
No enjoyment	Ref			Ref			Ref		
Mixed enjoyment	1.28	1.02, 1.6	0.031	1.27	0.97, 1.66	0.081	1.18	0.9, 1.55	0.226
Enjoyed school	1.64	1.33, 2.03	<0.001	1.48	1.16, 1.9	0.002	1.29	0.99, 1.67	0.057
Social class									
IV & V				Ref			Ref		
III Manual				1.18	1.03, 1.35	0.017	1.16	1.02, 1.33	0.028
III Non-manual				2.05	1.77, 2.37	<0.001	2.00	1.72, 2.31	<0.001
I & II				1.91	1.62, 2.25	<0.001	1.88	1.59, 2.21	<0.001
Maternal education									
CSE/vocational				Ref			Ref		
O-level				1.65	1.47, 1.86	<0.001	1.62	1.44, 1.82	<0.001
A-level				2.45	2.11, 2.84	<0.001	2.37	2.04, 2.75	<0.001
Degree				3.00	2.42, 3.73	<0.001	2.95	2.38, 3.66	<0.001
Cognitive ability				3.19	2.96, 3.43	<0.001	3.10	2.88, 3.34	<0.001
Female	1.50	1.38, 1.62	<0.001	1.80	1.62, 1.99	<0.001	1.72	1.55, 1.91	<0.001
Month of birth	0.97	0.96, 0.98	<0.001	0.95	0.93, 0.96	<0.001	0.95	0.93, 0.97	<0.001
Non-white	0.81	0.67, 0.97	0.024	0.86	0.53, 1.39	0.533	0.87	0.68, 1.12	0.275
School year									
2006/2007	Ref			Ref			Ref		
2007/2008	0.95	0.85, 1.06	0.378	0.95	0.82, 1.09	0.437	0.94	0.82, 1.09	0.413
2008/2009	0.94	0.79, 1.11	0.467	0.96	0.77, 1.18	0.672	0.94	0.76, 1.16	0.575
Age at Q1	0.75	0.53, 1.05	0.094	0.82	0.56, 1.22	0.331	0.85	0.57, 1.27	0.433
Age at Q2	0.79	0.51, 1.24	0.311	0.86	0.53, 1.39	0.533	0.88	0.54, 1.45	0.625

Home learning environment	1.07	1.03, 1.12	0.002
Likes teacher			
Not at all	Ref		
Sometimes	1.39	0.64, 3.01	0.399
Usually	1.33	0.64, 2.74	0.446
Always	1.55	0.75, 3.19	0.238
Temperament	1.03	0.99, 1.07	0.107
Work confidence	1.02	0.98, 1.06	0.4
Intelligence confidence	1.09	1.05, 1.13	<0.001
Friends score	0.98	0.96, 1.01	0.242

Supplementary Table 7: Logistic regression of GCSE points at age 16 in complete case sample. OR: Odds Ratio; CI: confidence interval; Ref: reference category; GCSE: General Certificate of Secondary Education; CSE: Certificate of Secondary Education; Q1: First enjoyment questionnaire at age 6; Q2: Second enjoyment questionnaire at age 6.5.

	Model 1: Unadjusted			Model 2: Family socioeconomic position			Model 3: IQ adjusted		
	OR	95% CI	p value	OR	95% CI	p value	OR	95% CI	p value
School enjoyment									
No enjoyment	Ref			Ref			Ref		
Mixed enjoyment	1.60	1.05, 2.43	0.029	1.88	1.16, 3.06	0.011	1.75	1.1, 2.78	0.019
Enjoyed school	2.42	1.67, 3.51	<0.001	2.39	1.55, 3.67	<0.001	2.15	1.41, 3.26	<0.001
Social class									
IV & V				Ref			Ref		
III Manual				1.25	0.93, 1.68	0.139	1.12	0.84, 1.49	0.43
III Non-manual				2.22	1.63, 3.01	<0.001	2.07	1.54, 2.79	<0.001
I & II				1.78	1.28, 2.47	0.001	1.62	1.18, 2.23	0.003
Maternal education									
CSE/vocational				Ref			Ref		
O-level				1.54	1.17, 2.02	0.002	1.58	1.22, 2.06	0.001
A-level				1.89	1.39, 2.56	<0.001	2.00	1.48, 2.68	<0.001
Degree				1.95	1.3, 2.91	0.001	2.06	1.39, 3.05	<0.001
Cognitive ability				3.05	2.67, 3.48	<0.001	3.02	2.66, 3.44	<0.001
Female	1.38	1.15, 1.65	<0.001	1.71	1.39, 2.1	<0.001	1.73	1.41, 2.12	<0.001
Month of birth	0.99	0.96, 1.02	0.415	0.96	0.93, 1	0.037	0.97	0.94, 1	0.074
Non-white	0.80	0.5, 1.3	0.377	0.65	0.38, 1.14	0.135	0.75	0.43, 1.3	0.301
School year									
2006/2007	Ref			Ref			Ref		
2007/2008	1.08	0.83, 1.39	0.577	0.99	0.74, 1.32	0.931	0.98	0.74, 1.3	0.895
2008/2009	1.02	0.7, 1.49	0.918	0.95	0.62, 1.46	0.819	0.99	0.66, 1.51	0.978
Age at Q1	0.64	0.29, 1.43	0.28	0.75	0.3, 1.85	0.529	0.66	0.28, 1.57	0.352

Age at Q2	1.38	0.57, 3.36	0.479	1.09	0.41, 2.87	0.868	1.04	0.41, 2.64	0.934
Home learning environment							1.14	1.04, 1.24	0.007
Likes teacher									
Not at all							Ref		
Sometimes							0.84	0.19, 3.7	0.823
Usually							1.08	0.26, 4.44	0.916
Always							1.11	0.27, 4.54	0.889
Temperament							1.03	0.97, 1.08	0.323
Work confidence							1.01	0.96, 1.06	0.731
Intelligence confidence							1.01	0.94, 1.09	0.819
Friends score							0.98	0.94, 1.02	0.374
Constant	0.96	0.58, 1.61	0.883	0.44	0.23, 0.85	0.014	0.10	0.02, 0.65	0.016

References

Royston, P., & White, I. (2011). Multiple Imputation by Chained Equations (MICE): Implementation in Stata. *Journal of Statistical Software*. https://doi.org/10.18637/jss.v045.i04
