# Supplementary material for: Associations between school enjoyment at age 6 and later educational achievement: evidence from a UK cohort study
Source: NPJ Sci Learn. 2021 Jun 15;6:18. doi: 10.1038/s41539-021-00092-w (PMC8206254; doi:10.1038/s41539-021-00092-w)
Supplement: Supplementary file 2 — Reporting Summary [file 41539_2021_92_MOESM2_ESM.pdf]

nature research

Corresponding author(s): Tim Morri

Last updated by author(s): Feb 1, 2021

Reporting Summary

Nature Research wishes to improve the reproducibility of the work that we publish. This form provides structure for consistency and transparency in reporting. For further information on Nature Research policies, see our Editorial Policies and the Editorial Policy Checklist.

Statistics

For all statistical analyses, confirm that the following items are present in the figure legend, table legend, main text, or Methods section.

n/a	Confirmed	
☐	☒	The exact sample size (n) for each experimental group/condition, given as a discrete number and unit of measurement
☐	☒	A statement on whether measurements were taken from distinct samples or whether the same sample was measured repeatedly
☐	☒	The statistical test(s) used AND whether they are one- or two-sided<br>*Only common tests should be described solely by name; describe more complex techniques in the Methods section.*
☐	☒	A description of all covariates tested
☐	☒	A description of any assumptions or corrections, such as tests of normality and adjustment for multiple comparisons
☐	☒	A full description of the statistical parameters including central tendency (e.g. means) or other basic estimates (e.g. regression coefficient) AND variation (e.g. standard deviation) or associated estimates of uncertainty (e.g. confidence intervals)
☐	☒	For null hypothesis testing, the test statistic (e.g. F, t, r) with confidence intervals, effect sizes, degrees of freedom and P value noted<br>*Give P values as exact values whenever suitable.*
☒	☐	For Bayesian analysis, information on the choice of priors and Markov chain Monte Carlo settings
☒	☐	For hierarchical and complex designs, identification of the appropriate level for tests and full reporting of outcomes
☐	☒	Estimates of effect sizes (e.g. Cohen's d, Pearson's r), indicating how they were calculated

Our web collection on statistics for biologists contains articles on many of the points above.

Software and code

Policy information about availability of computer code

Data collection	All code used to process and analyse the data are available at https://github.com/timtmorris/ALSPAC_school_enjoyment.
Data analysis	Stata version 16.

For manuscripts utilizing custom algorithms or software that are central to the research but not yet described in published literature, software must be made available to editors and reviewers. We strongly encourage code deposition in a community repository (e.g. GitHub). See the Nature Research guidelines for submitting code & software for further information.

Data

Policy information about availability of data

All manuscripts must include a data availability statement. This statement should provide the following information, where applicable:

- Accession codes, unique identifiers, or web links for publicly available datasets
- A list of figures that have associated raw data
- A description of any restrictions on data availability

The data that support the findings of this study are available from the ALSPAC study, but restrictions apply to the availability of these data, which were used under license for the current study, and so are not publicly available. The data are available subject to approval from the ALSPAC study Executive. The empirical dataset used in this study has been archived with the ALSPAC study under the project identifier B2193. For further information please see http://www.bristol.ac.uk/alspac/researchers/.

Field-specific reporting

Please select the one below that is the best fit for your research. If you are not sure, read the appropriate sections before making your selection.

☐ Life sciences ☒ Behavioural & social sciences ☐ Ecological, evolutionary & environmental sciences

For a reference copy of the document with all sections, see nature.com/documents/nr-reporting-summary-flat.pdf

Behavioural & social sciences study design

All studies must disclose on these points even when the disclosure is negative.

Study description	This study used data from a longitudinal cohort study, the Avon Longitudinal Study of Parents and Children (ALSPAC).
Research sample	Participants were children from ALSPAC. Pregnant women resident in Avon, UK with expected dates of delivery 1st April 1991 to 31st December 1992 were invited to take part in the study. These women, their children and their partners continue to contribute data to the ALSPAC study.
Sampling strategy	We used the full available ALSPAC sample, consisting of those who had completed questionnaires and opted into data linkage.
Data collection	Data were collected by ALSPAC using self-report questionnaires, parent-report questionnaires, direct assessment clinics, and data linkage.
Timing	Data collection for the ALSPAC study commenced in 1991 and is ongoing.
Data exclusions	Participants were excluded from the analyses study based upon missing month of birth, sex or ethnicity status.
Non-participation	Attrition from the ALSPAC study
Randomization	No randomisation was used.

Reporting for specific materials, systems and methods

We require information from authors about some types of materials, experimental systems and methods used in many studies. Here, indicate whether each material, system or method listed is relevant to your study. If you are not sure if a list item applies to your research, read the appropriate section before selecting a response.

Materials & experimental systems

n/a	Involved in the study	
☒	☐	Antibodies
☒	☐	Eukaryotic cell lines
☒	☐	Palaeontology and archaeology
☒	☐	Animals and other organisms
☐	☒	Human research participants
☒	☐	Clinical data
☒	☐	Dual use research of concern

Methods

n/a	Involved in the study	
☒	☐	ChIP-seq
☒	☐	Flow cytometry
☒	☐	MRI-based neuroimaging

Human research participants

Policy information about studies involving human research participants

Population characteristics	See above.
Recruitment	Pregnant women resident in Avon, UK with expected dates of delivery 1st April 1991 to 31st December 1992 were invited to take part in the study.
Ethics oversight	ALSPAC Law and Ethics Committee.

Note that full information on the approval of the study protocol must also be provided in the manuscript.
